# The effect of the human cumulus cells-conditioned medium on in vitro maturation of mouse oocyte: An experimental study

**DOI:** 10.18502/ijrm.v18i12.8023

**Published:** 2020-12-21

**Authors:** Maryam Adib, Seyed Morteza Seifati, Mahmood Dehghani Ashkezari, Arezoo Khoradmehr, Roshan Rezaee-Ranjbar-Sardari, Somayyeh Sadat Tahajjodi, Behrouz Aflatoonian

**Affiliations:** ^1^Medical Biotechnology Research Center, Ashkezar Branch, Islamic Azad University, Ashkezar, Yazd, Iran.; ^2^Stem Cell Biology Research Center, Yazd Reproductive Sciences Institute, Shahid Sadoughi University of Medical Sciences, Yazd, Iran.; ^3^Research and Clinical Center for Infertility, Yazd Reproductive Sciences Institute, Shahid Sadoughi University of Medical Sciences, Yazd, Iran.; ^4^Department of Reproductive Biology, School of Medicine, Shahid Sadoughi University of Medical Sciences, Yazd, Iran.; ^5^Department of Advanced Medical Sciences and Technologies, School of Paramedicine, Shahid Sadoughi University of Medical Sciences, Yazd, Iran.

**Keywords:** Germinal vesicle, Cumulus cell, Conditioned medium, In vitro fertilization, In vitro maturation, Oocyte.

## Abstract

**Background:**

To increase the results of infertility treatment, many efforts have been made to improve the treatment methods. As assisted reproductive technology is mainly using cell culture methods, one of the approaches to improve this technology is conditioned medium from different sources. It is desirable to apply *in vitro* maturation (IVM) and use oocytes from normal cycles instead of stimulating ovulation.

**Objective:**

To investigate the effect of human cumulus cell condition medium (hCCCM) on the IVM of immature mouse oocytes and morphology.

**Materials and Methods:**

In this experimental study, 240 germinal vesile oocytes were collected from four-six wk-old mice after 48 hr of 5IU pregnant mare serum gonadotropin (PMSG) injection and cultured in hCCCM (test group, n = 120) and DMEM + 20% FBS (control group, n = 120). The IVM rates and changes in perivitelline space (PVS) and shape were investigated at 8, 16, and 24 hr following the culture. The mature (MII) oocytes were subjected to in vitro fertilization (IVF) and the fertilization rate was assessed in three days.

**Results:**

A significant difference was observed between the maturation rates in the hCCCM and control groups (24.16% vs 0%; p = 0.001), as well as morphologic changes between the two groups (p = 0.04, p = 0.05). The development rate for MII oocytes attained from IVM in the hCCCM group was 27.58% (2-cell) and 6.89% (4-cell). Data displayed that hCCCM is an effective medium for oocytes maturation compared to the control medium.

**Conclusion:**

hCCCM supports oocyte *in vitro* growth and maturation. Moreover, hCCCM changes the oocyte shape and size of perivitelline space.

## 1. Introduction

An important part of reproductive medicine in infertility treatment is ovarian stimulation by drugs to gain a higher number of mature oocyte retrieved for the purpose of increasing pregnancy rates in comparison with the normal cycles (1). Recently, drug usage has been founded to make final oocyte maturation in patients. In this case, one of the concerns is the oocyte hyper stimulation syndrome (OHSS) that can cause a significant injury in women (2). *In vitro* maturation (IVM) is a desirable alternative method which has already gained successful results in supporting a natural *in vitro* fertilization (IVF) cycle treatment with immature oocyte retrieval. If such a technique develops further to improve the number and quality of IVM outcomes, in future, this might be replaced with conventional stimulation approaches using drugs and hormones (3).

The major advantage of applying IVM is to get rid of the gonadotropin drugs and the risk of OHSS in female patients (4). One of the problems in the IVM process is providing an effective medium that mimics *in vivo* conditions for the growth and development of immature oocytes (5, 6). Oocyte development is facilitated in the presence of several growth factors (GFs) and specific growth signals that are secreted by the cumulus cells (7). Cumulus cells are considered as the oocyte nursing cells during oogenesis which provides a suitable microenvironment (niche) containing GFs and other components required for oogenesis (8-12). New studies have shown that the cumulus cell-conditioned medium (CCCM) can meaningfully affect the IVM and IVF rates (8, 9). Furthermore, it has been shown that the CCCM can be used as an *in vitro* niche for female gamete development from pluripotent stem cells in buffalo (11) and human (12). Also, a recent study has revealed that 50% human CCCM (hCCCM) for 21 days can direct the differentiation of peritoneum mesenchymal stem cells into the ovarian cell-like cells and increase the expression of granulosa cells and female germ cell genes and markers (13).

Recently, the effect of the CCCM and granulusa cell conditioned medium (GCCM) on mice primordial follicles development and growth was assessed in a new study. Their results have indicated that CCCM and GCCM play essential roles in oocyte maturation by secreting supporting GFs inside the medium (10, 13, 14). Thereby, we assumed that hCCCM may have an effective and supportive impact in improving IVM. The present study is the first report to investigate whether hCCCM collected from cumulus cells in an adherent culture (15) can improve the IVM in mice or not. The practical goals of our study are: Laboratory maturation optimization of immature oocytes using conditioned environments, use of factors in conditioned medium (CM) in the treatment of early menopause, and prevention of OHSS by harvesting germinal vesicle (GV) without human chorionic gonadotropin (hCG) injection.

## 2. Materials and Methods

### Study design and materials

In this experimental study, chemicals and reagents were purchased from Sigma Aldrich Co. (UK). Culture media and supplements were purchased from Invitrogen Co. (UK), unless otherwise stated. This study was conducted in the Biotechnology Department of Yazd Infertility Research and Treatment Center in 2018-2019. The input criteria in this study were immature oocytes of mice and the output criteria were mature (MII) oocytes in test and control media.

### Preparation of hCCCM 

The CCCM was collected from growing human cumulus cells in the adherent monolayer culture condition as explained elsewhere (15). After the oocyte retrieval, cumulus cells were mechanically separated from cumulus-oocyte complexes (COCs) using syringe needles following enzymatic treatment. Following washing, cumulus cells were pelleted by centrifugation for 10 min at 500 g. Then, they were cultured with Dulbecco's Modified Eagle Medium + 20% Fetal bovine serum (DMEM + 20% FBS) (Invitrogen, Gibco, UK) medium. The medium from cultures was collected every three days after subculture with 80% confluency. The CCCM was filtered and stored at -20°C (15).

### Animals

Oocytes were obtained from the ovaries of 6-8 wk-old Naval Medical Research Institute (NMRI) female mice, while testes were obtained from 6-8 wk-old NMRI male mice. These mice were housed and bred in the animal house at the Biotechnology Unit of the Yazd Reproductive Sciences Institute. The mice were kept in a 12 hr light/dark cycle, a temperature range of 22-25°C, humidity 40-60%, and enough nutriment.

### Immature oocytes collection and their IVM

**Figure 1:**
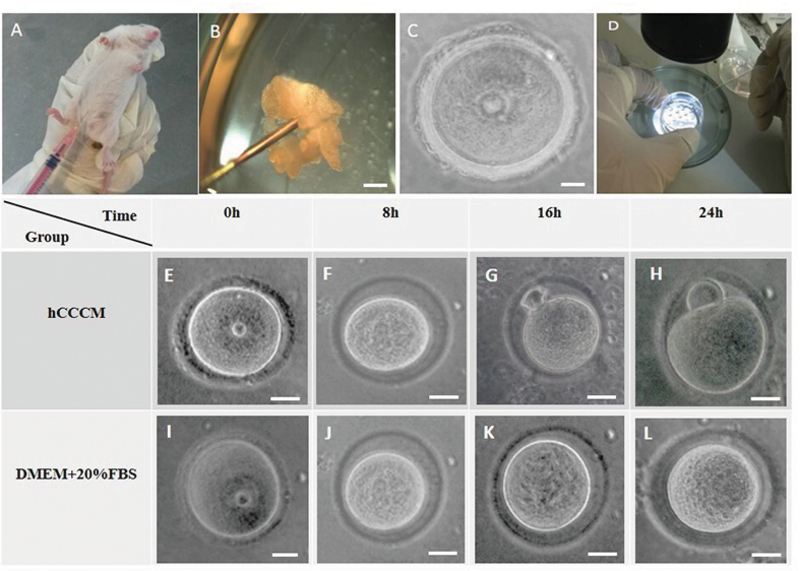
GV oocytes retrieval process in mice and IVM. First, an injection of 5IU PMSG was given to four-six week-old mouse (A). Ovaries were taken from stimulated mice and using the stereo microscope ovary was scratched (B). Each immature GV oocyte (C) was recovered and then cultured separately and individually in 10-μl micro drops (D). IVM progress stages at different times in the hCCCM and control groups. After 16 and 24 hr in the hCCCM medium, some of the GV oocytes (E) developed and became MI (F) and mature MII oocytes (G, H). Nevertheless, in the DMEM + 20% FBS medium, GV oocytes (I) could develop to MI oocytes (J-L) but none of them could complete the IVM process to form MII oocytes.

Five IU pregnant mare serum gonadotropin (PMSG) was injected into four-six wk-old female mice (Figure 1A). GV oocytes from the female mice ovaries were retrieved 48 hr after the injection by the scratching method on the ovaries using sterile 28-gauge needles under a stereomicroscope (Olympus, Japan; Figures 1B, 1C, and 1D). GV oocytes (Figure 1C) were single cultured within the separated individual microdrops of hCCCM and DMEM + 20% FBS as the test and control groups, respectively. The control group of this study is similar to the control of our previously published study (16). In total, 240 immature GV oocytes were used in this study (n= 120/each). Next, they were placed in an incubator (37°C temperature, 5% CO${}_{2}$). Oocyte maturation, shape, and perivitelline space (PVS) changes were evaluated once every eight hours by a stereomicroscope. Oocytes with a polar body were considered as MII oocytes and were selected for the IVF procedure.

### IVF and embryo development

The developmental potential rate of MII oocytes was evaluated by IVF. Male mice spermatozoa cells were obtained from the caudal epididymis and capacitated for 1 hr at 37°C. MII oocytes were incubated with sperms in the GIVF medium (Vitrolife, Sweden) for 4 hr. After washing the oocytes for removal of extra sperms, they were cultured in a G1-plus micro-drop (Vitrolife, Sweden) and placed in an incubator (37°C temperature, 5% CO${}_{2}$), for three days. The embryo development rate was evaluated at 24, 48, and 72 hr after the insemination using a stereomicroscope.

### Ethical consideration

The research proposal was approved by Shahid Sadoughi University of Medical Sciences ethics committee (code: IR.SSU. REC.1397.087). In this study, cumulus cells were obtained from the male-factor infertile couples participated in our previous study (15) according to the approval of the ethics committee. All ethical protocols for working with laboratory animals were observed in this study.

### Statistical analysis

Changes in oocytes' shape and PVS, maturation rate and embryo development rate in mice oocytes were assessed at each step and compared between the two groups. Statistical analysis was done using the Chi-square test with an R.V.16 software. P ≤ 0.05. was considered as statistically significant.

## 3. Results

### Effect of hCCCM on IVM 

Resumption of meiosis to the MII stage or IVM of immature GV oocytes (Figures 1E and 1I) was evaluated after 8 hr (Figures 1F and 1J), 16 hr (Figures 1G and 1K), and 24 hr (Figures 1H and 1L) following the oocyte pick-up and culture in the test (hCCCM) and control (DMEM + 20% FBS) groups. Significant difference was seen in the IVM rates between the hCCCM and control medium at 16 and 24 hr (p = 0.001, Table I). Table I shows the number and percentage of degenerated, meiosis I stage (MI), and MII oocytes in the test and control groups at different times. Some of the immature oocytes were developed to the MI stage after 8 hr in both media. While the rate of the degenerated oocytes at 8 hr in the hCCCM medium was less than the control medium (Table I), the rate of MI oocytes in the hCCCM group was significantly higher than the control group (p = 0.01; Table I). The percentage of the MII oocytes in the hCCCM medium at 8 hr was similar to the control medium (Table I).

IVM rate was checked in both groups after 16 hr and as a result, the degenerated oocytes in the hCCCM group were less than degenerated oocytes in the control group (Table I). A percentage of MI and MII oocytes in hCCCM was higher than the control group (Table I). The degenerated oocytes after 24 hr in the hCCCM were less than the degenerated oocytes in the control group (Table I). Half of the oocytes developed to the MI stage in hCCCM after 24 hr (Table I) and about one-fourth of them reached to the MII stage (Table I); however, in the control group, only 9 of the 120 oocytes developed further to the MI stage and none of them developed to the MII (Table I).

Overall, data showed that the degeneration rate in both groups increased after 24 hr, and although this ratio was slightly lower than the control group, the difference was not significant (p = 0.46; Table I). In both media, the immature GV oocytes supported the MI stage which this progress was higher in the hCCCM medium (Table I). The MI stage decreasing trend in the hCCCM medium during 24 hr might be because of the growth of the oocytes to the MII stage (Table I). Also, in the control medium, the MI rate decreased after 24 hr which was either due to the degeneration or arrest after 16 hr (Table I). The MII rate increase in the test group after 24 hr shows the positive effect of the time on oocyte growth in the hCCCM group. In the control medium, at 24 hr, despite the growth of the oocytes MI stage, none of them reach to the MII stage (Table I). Our results show that there are factors secreted from human cumulus cells (hCCs) that support IVM in mice.

### Morphology evaluation of oocytes

**Figure 2:**
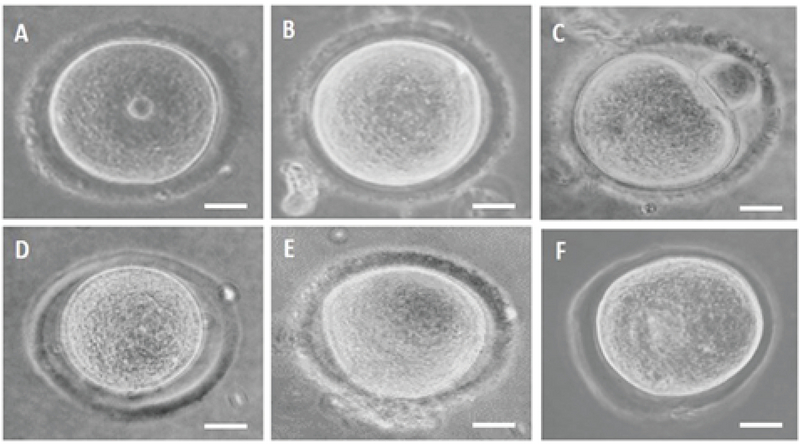
The PVS and shape change rate of oocytes in the hCCCM and control media during the IVM process. (A) Normal GV. (B) Normal MI. (C) Normal MII. (D) Large PVS. (E and F) Irregular shape and large PVS.

Morphological assessment is one of the important and basic tools for evaluating the quality of oocytes (16, 17). In this study, large PVS and irregular shape of oocytes were checked as the morphological criteria. Figure 2A-2F show examples of normal and abnormal oocytes in terms of shape and PVS. The rates of large PVS oocytes and rates of irregular-shape oocytes at 8, 16, and 24 hr in the hCCCM medium and control group were shown in the table II. The results of this study show that there are significant differences in the rate of large PVS (p = 0.05) and irregular-shaped (p = 0.04) between the hCCCM and control groups (Table II).

### Embryo development rate following IVF 

**Figure 3:**
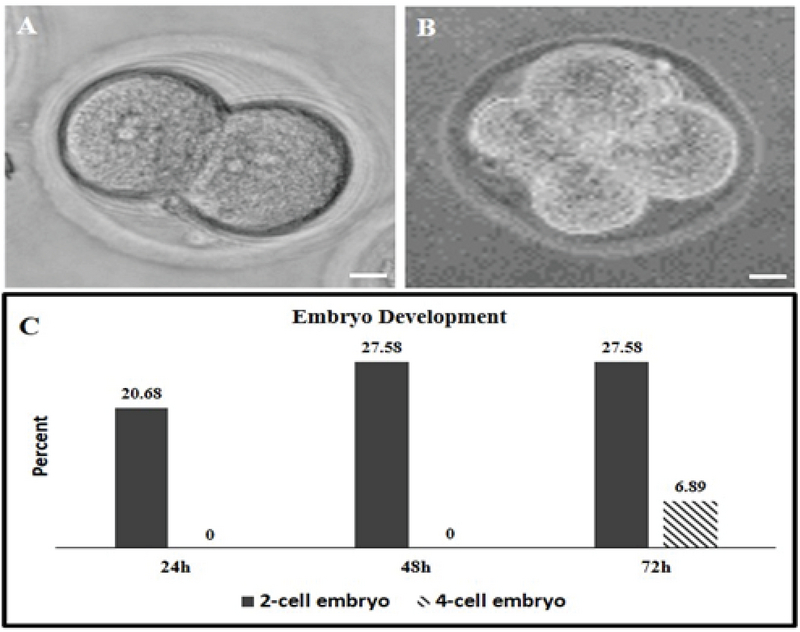
Embryo development following IVF of MII oocytes from the hCCCM group. Following IVF from 29 oocytes, after 24 hr, six of the fertilized oocytes developed to two-cell embryos (A and C) and none of them reached four cells. After 48 hr, eight embryos developed to two cells (A and C) and none of them reached four-cells embryos. At 72 hr following IVF, eight embryos arrested at two-cell stage and instead two embryos were observed at four-cell stage (B and C). The embryos did not follow further but after three days none of them formed blastocyst.

MII oocytes were inseminated for the IVF procedure to evaluate their fertilization capacity after IVM. Following IVF, embryo development was checked for three days. None of the embryos were developed further than four cells in the cleavage stage (Figures 3A and 3B). The percentage of two-cell and four-cell embryos after 24, 48, and 72 hr were shown in the Figure 3C. The developmental rate of embryos to two-cell stage increased until the second day post IVF, then stopped on the third day (Figure 3C), and the formation of the four-cell embryos began from the third day post IVF (Figure 3C).

**Table 1:** IVM rates at 8, 16, and 24 hr in the hCCCM and DMEM + 20% FBS groups


**Group**	**Deg.**			**MI**			**MII**		
	**8 hr**	**16 hr**	**24 hr**	**8 hr**	**16 hr**	**24 hr**	**8 hr**	**16 hr**	**24 hr**
**hCCCM**	21 (17.5)	28 (23.33)	31 (25.83)	87 (72.5)	69 (57.5)	60 (50)	0 (0)	23 (19.16)	29 (24.16)
**DMEM + 20%FBS**	29 (24.17)	40 (33.33)	63 (52.5)	11 (9.17)	9 (7.5)	9 (7.5)	0 (0)	0 (0)	0 (0)
**P-value**	0.46			* 0.01			*0.001		
Data presented as n (%) in different times. Chi-square test; *Significant level of ≤ 0.05, hCCCM: Human cumulus cell-conditioned medium; DMEM + 20%FBS: Dulbecco's modified eagle's medium + 20% fetal bovine serum; Deg.: Degenerated; MI: Meiosis I; MII: Meiosis II									

**Table 2:** Rate of oocytes large/normal PVS and irregular/normal shape following IVM in hCCCM and control group at different times


**Group**	**hCCCM**			**DMEM + 20% FBS**			**P-value**
	**8 hr**	**16 hr**	**24 hr**	**8 hr**	**16 hr**	**24 hr**	
**Large PVS **	41 (34.16)	47 (39.16)	57 (47.5)	10 (8.33)	29 (24.16)	35 (29.16)	* 0.05
**Irregular shape **	19 (15.83)	28 (23.33)	38 (31.66)	8 (6.66)	31 (25.83)	51 (42.5)	* 0.04
Data presented as n (%) in different times. Chi-square test; *Significant level of ≤ 0.05, hCCCM: Human cumulus cell-conditioned medium; DMEM + 20% FBS: Dulbecco's modified eagle's medium + 20% fetal bovine serum; PVS: Perivitelline space							

## 4. Discussion

This study assessed the effect of the hCCCM on the IVM rates of the GV oocytes in mice. The data of this study show that hCCCM supports IVM of mouse GV oocytes compared to the DMEM + 20% FBS as the control group (24.16% vs 0%). Additionally, the MII oocytes achieved by IVM in the hCCCM medium were fertilized in vitro (IVF) from which 6.89% developed to the four-cell stage. Usually, 70-85% of oocytes from stimulated cycles are MII stage, with the remainder variably at GV or MI stage (17).In some women, many immature oocytes (> 30%) can be produced despite the use of contemporary stimulation for clinical IVF. In such people, the fertilization, and embryo development rates are usually low (18, 19).

Thus, many researches were led based on the choice of proper factors to adapt the culture condition to progress the oocyte IVM efficacy in mouse, bovine, and human (20-22). It was shown that with the addition of some GFs, for example, Epidermal Growth Factors (EGFs) or insulin Growth Factors (IGFs), to the IVM medium, the rate of maturity and embryo development can be improved considerably (23, 24). The supportive effect of the EGF was shown in several species such as canine (25), mouse (26), and humans (27). Also, it was revealed that growth differentiation factor 9 (GDF9) supports the folliculogenesis in bovine (28), mice (29), and human (30). Additionally, CM is a vital culture complement device in the IVM method (28, 31). Similar to our study, cross-species experiments have shown the CM positive effects of one species in the IVM of another species' oocytes (28, 31). For example, the IVM of canine oocytes in bovine COC-CM (9) or canine oocyte IVM in the CM of mouse embryonic fibroblasts (32), IVM of mouse oocytes in the CM of human bone marrow mesenchymal stem cell (33), and mouse oocyte IVM in the CM of the human testicular cell (16). It was shown that the CM of granulosa cells facilitate IVM as confirmed by the MII oocytes formation and gene expression profile assessment (31).

Our data confirms the previously reported data as hCCCM supports the IVM in mice oocytes. Interestingly, the data reveals that factors secreted by CCs during the first eight hours of maturation are vital for oocyte development. Our data supports the previous report which indicated that the first eight hours of maturation is vital for the achievement of oocyte growth during the remaining 15 hr of maturation (8). Using metabolomics profile, it was shown that the IVM of denuded oocytes in COC-CM resulted in a better outcome than the IVM of denuded oocytes that completed maturation in standard IVM medium. Cumulus cells are necessary for the growth of oocytes during maturation and are supposed to secrete several factors that improve the growth ability of denuded oocytes (8). In another study, the short-term exposure of COCs to the IVM medium prior to ICSI indicated the improvement of the rate of the MII oocyte and embryo development (34). Thus, CCCM has the elements that play a critical role in oocyte maturation, which can be used to develop a new condition to improve IVM results.

The GV retrieval techniques and the IVM basal medium are additional topics that may have an influence on the result of IVM (35). Here, we have used the scratching method for oocyte retrieval and DMEM + 20% FBS was used as a basal medium for both the control and test groups to keep the condition as consistent as possible during the study. The other issue which was investigated in the current study was the effect of the medium (test and control) on the morphology of the oocytes following culture. Some data have shown that oocyte morphology plays a significant role in their subsequent fertilization and cleavage rates and on the embryo quality (36, 37). Moreover, IVM of oocytes without anomalies produces higher-quality embryos than eggs with anomalies (36). Irregularities of PVS are among the most important anomalies of the extra cytoplasmic cause. It has been studied that a large PVS might be associated with increased degeneration of oocytes (36) and lower fertilization rates (37). In the current study, hCCCM caused the formation of large PVS in IVM oocytes, whereas the irregular shape of the oocytes was observed more in the control group. The reasons for these differences are unknown but might be due to the osmolality of the different IVM cultures and factors within the hCCCM.

## 5. Conclusion

In conclusion, for the first time, our data indicated that hCCCM from cultures of adherent cumulus cells can support IVM of mouse GV oocytes. The IVF/IVC of the MII oocytes was assessed for three days to the formation of two- and four-cells embryos. Moreover, the hCCCM caused the formation of large PVS, however, the shape of oocytes became more irregular in the DMEM + 20% FBS.

##  Conflict of Interest

The authors declare no conflicts of interests in with regard to the present study.
